# Supplementary material for: Live tracking of moving samples in confocal microscopy for vertically grown roots
Source: eLife. 2017 Jun 19;6:e26792. doi: 10.7554/eLife.26792 (PMC5498147; doi:10.7554/eLife.26792)
Supplement: Supplementary file 1. — (1) Board design of the illumination system. (2) Mounting plate for Axio Observer. (3) Modification of the laser safety. (4) Rotation stage inset. DOI: http://dx.doi.org/10.7554/eLife.26792.021 [file elife-26792-supp1.zip › SupplementalFile1/Modification of the laser safety.docx]

Modification of the laser safety:

Since the transmitted light arm can no longer be reclined when the microscope is turned 90° access to the sample is hindered by the laser safety shield. To restore access the laser safety was modified in the following way:

The reed switch (arrow) located at the base of the transmitted light arm was removed. The screws holding the safety sheet were replaced by pins and magnets were fitted onto the shield. The reed switch was relocated to the bracket that holds the shield.


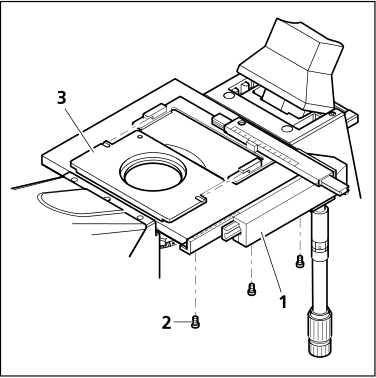


This way the shield can be easily removed without the need for tools and the reed switch closes only when the shield is put back into place maintaining laser safety.


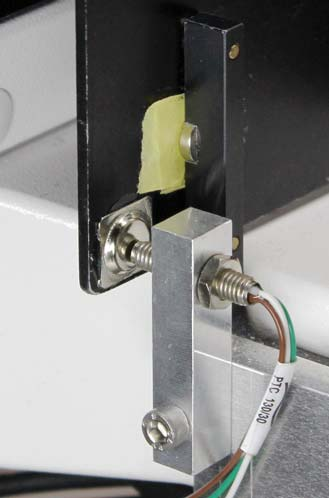


Detailed view of the modified safety shield and reed switch.
