# Supplementary material for: Live tracking of moving samples in confocal microscopy for vertically grown roots
Source: eLife. 2017 Jun 19;6:e26792. doi: 10.7554/eLife.26792 (PMC5498147; doi:10.7554/eLife.26792)
Supplement: Supplementary file 2. — (1) Implementation of TipTracker on two commercial platforms (Zeiss LSM700 and LaVisionBiotec TriMScopeII) and a short manual how to use it. (2) Fiji macros to convert LSM files into Hyperstacks. (3) Collection of simple AutoIt scripts and description on how to adapt them to a specific setup. (4) Script to calculate a post-rotation position list to use with the rotation stage. DOI: http://dx.doi.org/10.7554/eLife.26792.022 [file elife-26792-supp2.zip › SupplementalFile2/RotationStage/HOW TO USE THE ROTATION STAGE.rtf]

1.	put disk with hole in holder
2.	reuse center.czi
3.	move hole into center of field of view
4.	add to position list 
5.	save position list as "center.pos"
6.	delete from position list
7.	swap disk with your sample
! steps 1-7 only need to be performed after a complete shut-down of the entire system
 
8.	define your sample position list 
9.	save position list
10.	run experiment part I 
11.	rotate stage 90 degree counter clockwise
12.	start "rotatepositionlist.m"
13.	load 90positionlist.pos
14.	run experiment part II

! never use "set as zero"
