# Supplementary material for: Live tracking of moving samples in confocal microscopy for vertically grown roots
Source: eLife. 2017 Jun 19;6:e26792. doi: 10.7554/eLife.26792 (PMC5498147; doi:10.7554/eLife.26792)
Supplement: Supplementary file 2. — (1) Implementation of TipTracker on two commercial platforms (Zeiss LSM700 and LaVisionBiotec TriMScopeII) and a short manual how to use it. (2) Fiji macros to convert LSM files into Hyperstacks. (3) Collection of simple AutoIt scripts and description on how to adapt them to a specific setup. (4) Script to calculate a post-rotation position list to use with the rotation stage. DOI: http://dx.doi.org/10.7554/eLife.26792.022 [file elife-26792-supp2.zip › SupplementalFile2/TipTrackerZeissLSM700/AutoItScripts/ZEN software screenshot.pdf]

Supplemental Figure

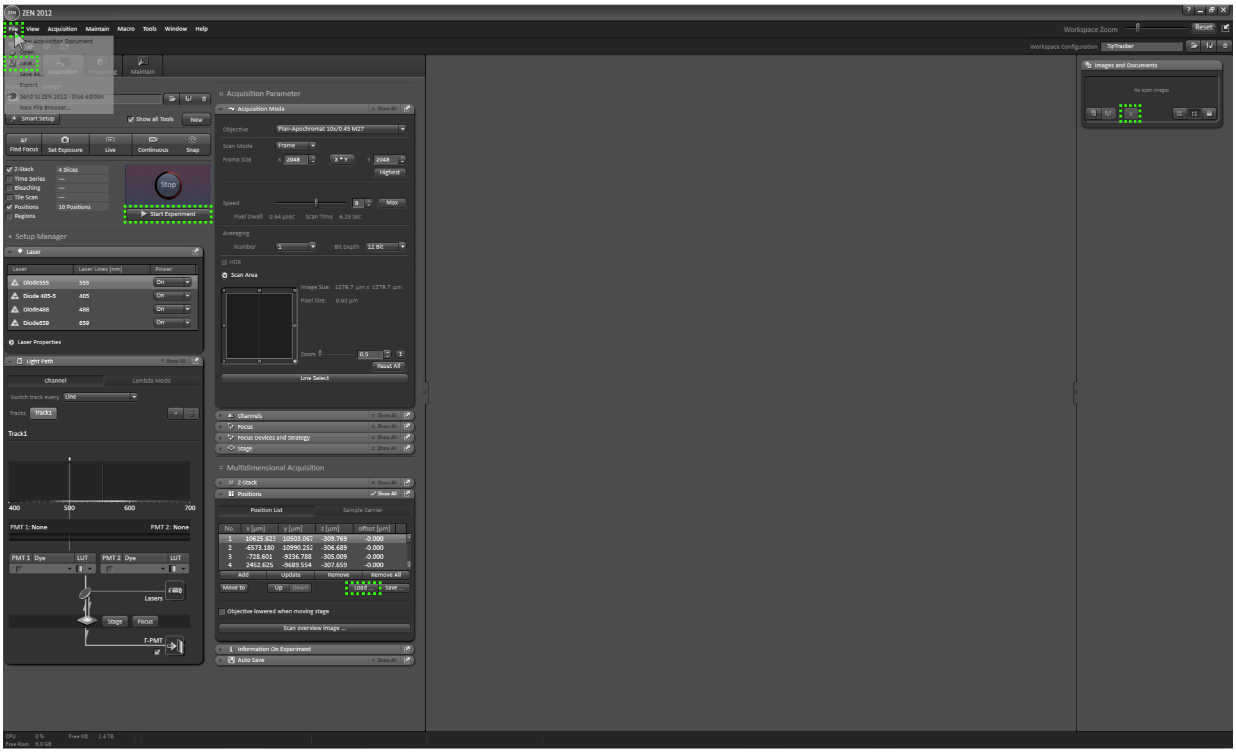

Screenshot of the Zen Graphical User Interface. The user has to specify screen coordinates of the buttons that are needed for loading a position list, starting the experiment and saving and closing the resulting image. These buttons are highlighted in green dashed boxes for the Zeiss Zen graphical user interface. The AutoIt software will use these coordinates to interface the TipTracker with the image acquisition software.
