# Supplementary material for: Live tracking of moving samples in confocal microscopy for vertically grown roots
Source: eLife. 2017 Jun 19;6:e26792. doi: 10.7554/eLife.26792 (PMC5498147; doi:10.7554/eLife.26792)
Supplement: Supplementary file 2. — (1) Implementation of TipTracker on two commercial platforms (Zeiss LSM700 and LaVisionBiotec TriMScopeII) and a short manual how to use it. (2) Fiji macros to convert LSM files into Hyperstacks. (3) Collection of simple AutoIt scripts and description on how to adapt them to a specific setup. (4) Script to calculate a post-rotation position list to use with the rotation stage. DOI: http://dx.doi.org/10.7554/eLife.26792.022 [file elife-26792-supp2.zip › SupplementalFile2/TipTrackerZeissLSM700/Manual/shortManual.docx]

1. Set up experiment as usual (don't use rotation, don't set up time series)
2. Save the position list into a new folder
3. Close all images, load workspace TipTracker, check 'show all' @ positions, uncheck 'show all' in scan field, maximize ZEN window
4. At this point the screen should look exactly like: “howZENshould look like.jpg”
5. Start TipTracker
6. Load position list into TipTracker
7. Define output folder, image names, maximum allowed shift
8. Set up time series
9. Press 'Start' and let go of mouse
10. Watch first 3 cycles if the shift is calculated correctly. If not 'kill'

After the experiment has finished:

1. Use supplied Fiji macro (“convertLSM2hyperstack.ijm”) to convert data set into single position hyperstacks
2. Stabilize stacks further (FIJI stack reg or my matlab image-stabilizer)
3. Also look at growth rate data output
