# Supplementary figures and images for: Live tracking of moving samples in confocal microscopy for vertically grown roots
Source: eLife. 2017 Jun 19;6:e26792. doi: 10.7554/eLife.26792 (PMC5498147; doi:10.7554/eLife.26792)

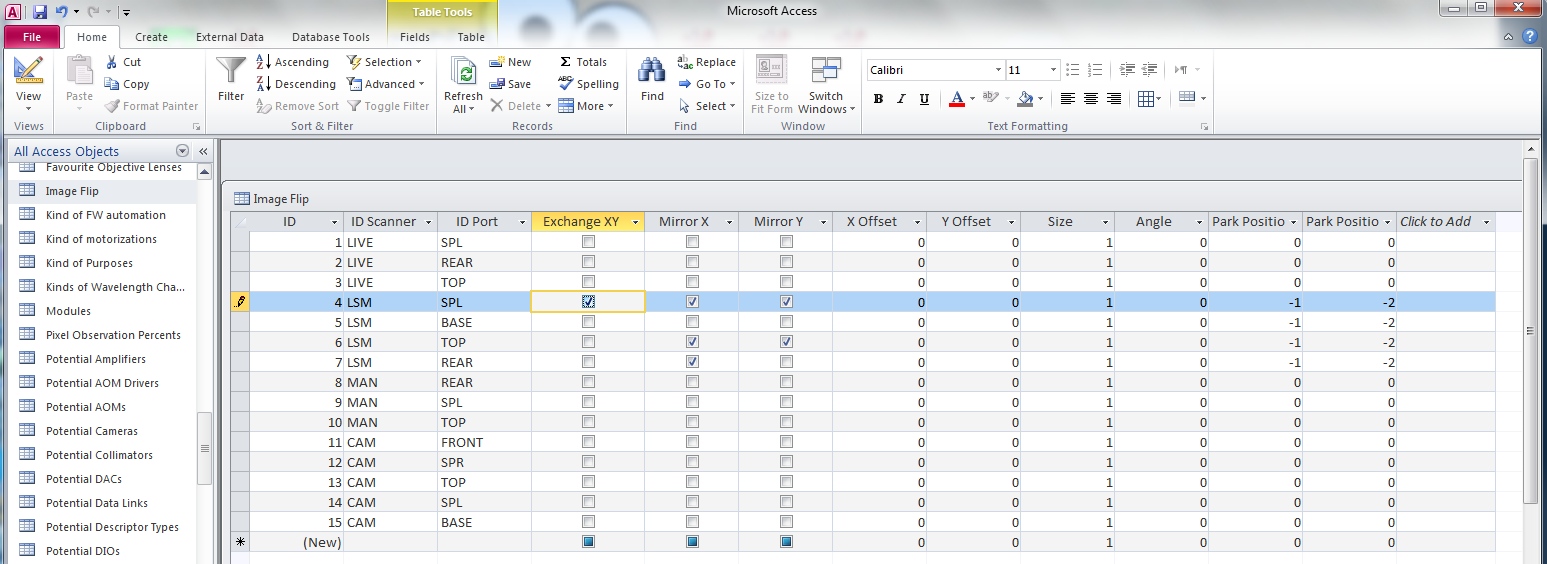

Supplement: Supplementary file 2. — (1) Implementation of TipTracker on two commercial platforms (Zeiss LSM700 and LaVisionBiotec TriMScopeII) and a short manual how to use it. (2) Fiji macros to convert LSM files into Hyperstacks. (3) Collection of simple AutoIt scripts and description on how to adapt them to a specific setup. (4) Script to calculate a post-rotation position list to use with the rotation stage. DOI: http://dx.doi.org/10.7554/eLife.26792.022 [file elife-26792-supp2.zip › SupplementalFile2/TipTrackerZeissLSM700/DatabaseChanges/ImageFlip.jpg]

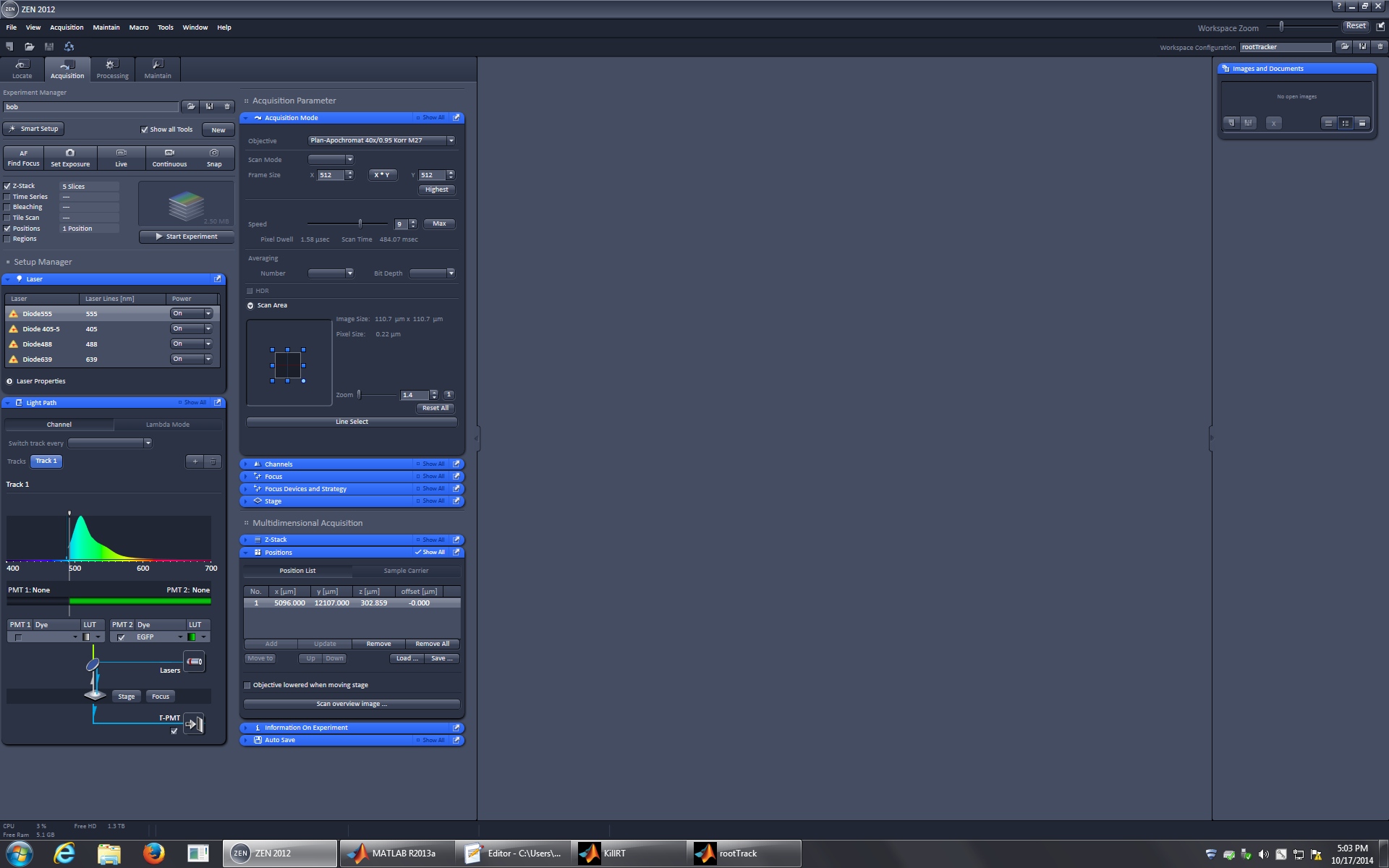

Supplement: Supplementary file 2. — (1) Implementation of TipTracker on two commercial platforms (Zeiss LSM700 and LaVisionBiotec TriMScopeII) and a short manual how to use it. (2) Fiji macros to convert LSM files into Hyperstacks. (3) Collection of simple AutoIt scripts and description on how to adapt them to a specific setup. (4) Script to calculate a post-rotation position list to use with the rotation stage. DOI: http://dx.doi.org/10.7554/eLife.26792.022 [file elife-26792-supp2.zip › SupplementalFile2/TipTrackerZeissLSM700/Manual/howZENshould look like.jpg]
